# The Effect of Cyclic Ozonation on the Molecular Stability of Fatty Acids and Cholesterol in Egg Yolk During Storage

**DOI:** 10.3390/molecules31193512

**Published:** 2026-10-02

**Authors:** Maciej Balawejder, Tomasz Piechowiak, Zofia Sokołowicz, Jacek Malec, Radosław Józefczyk, Izabela Szczepanik, Natalia Matłok

**Affiliations:** 1 Faculty of Technology and Life Science, Department of Chemistry and Food Toxicology, University of Rzeszow, 1a Ćwiklinskiej Street, 35-601 Rzeszów, Poland; mbalawejder@ur.edu.pl (M.B.); tpiechowiak@ur.edu.pl (T.P.); rjozefczyk@ur.edu.pl (R.J.); izabelaszczepanik7@gmail.com (I.S.); 2 Faculty of Technology and Life Sciences, Department of Animal Production and Poultry Products Evaluation, Institute of Food Technology and Nutrition, University of Rzeszow, 35-959 Rzeszow, Poland; zsokolowicz@ur.edu.pl; 3 Medical Post-Secondary School, Warzywna 1, 35-310 Rzeszów, Poland; jacek.malec1@gmail.com; 4 Faculty of Technology and Life Sciences, Department of Food and Agriculture Production Engineering, University of Rzeszow, Street Zelwerowicza 4, 35-601 Rzeszów, Poland

**Keywords:** egg quality, gaseous ozonation, lipid profile, docosahexaenoic acid (DHA), polyunsaturated fatty acids (PUFAs), cholesterol stability, eggshell barriers, lipid oxidation

## Abstract

Egg yolks are a valuable source of fatty acids and cholesterol; however, their lipid fraction undergoes natural degradative changes during storage. This study evaluated the effect of cyclic gaseous ozonation (5 ppm for 10 min, applied once weekly) on the fatty acid profile and cholesterol content of hen egg yolk during 5 weeks of storage. Chromatographic analyses (GC-MS) were conducted at weekly intervals (Weeks 0–5). In the control group (without ozonation), storage duration exerted a statistically significant effect on the lipid profile, leading to a progressive decline in the relative proportion of polyunsaturated fatty acids (PUFAs). Specifically, the linoleic acid content decreased from 8.88% to 5.6% by Week 5, while docosahexaenoic acid (DHA) underwent degradation to trace levels. Conversely, the ozonated group showed no statistically significant differences in the relative content of any of the thirteen identified fatty acids over the 5-week period, confirming the relative preservation and stability of the lipid profile. Cholesterol content remained stable (initial value 16.89 mg g^−1^) and was not significantly altered in either group throughout the storage period. These results indicate that cyclic gaseous ozonation represents an effective approach for preserving the relative stability of the yolk fatty acid profile (including DHA) without adversely affecting cholesterol levels, while the direct evaluation of primary oxidation indices remains a subject for future research.

## 1. Introduction

Hen eggs represent one of the most complete and nutritionally valuable food products in the human diet. They are a rich source of highly digestible protein, fat-soluble vitamins (A, D, E, and K), minerals, and valuable yolk lipids, including phospholipids and unsaturated fatty acids [1,2]. From a nutritional perspective, a particularly crucial role is played by the essential unsaturated fatty acids present in the yolk, such as linoleic acid (C_18:2_) and long-chain n-3 polyunsaturated fatty acids (PUFAs), notably docosahexaenoic acid (DHA), which exert beneficial effects on the human cardiovascular and nervous systems [3]. Egg yolk is also characterised by the presence of cholesterol, which performs an important structural role in cell membranes; nevertheless, the high concentration of unsaturated lipids within this matrix increases its susceptibility to adverse biochemical changes during storage. The high content of unsaturated fatty acids possessing reactive double bonds renders the egg yolk a system highly sensitive to degradative and oxidative processes occurring during distribution and storage [4,5,6]. The natural ageing of table eggs is associated with a series of interrelated physicochemical transformations. The primary phenomenon is the loss of carbon dioxide (CO_2_) through the micropores of the mineral shell, resulting in a rapid increase in albumen pH from an initial level of 7.6 to over 9.5 [7,8]. Such high alkalinity induces the depolymerisation of the ovomucin–lysozyme complex, leading to the thinning of the dense albumen gel and weakening the structural integrity of the vitelline membrane [7,9]. These alterations facilitate water migration between fractions and stimulate the diffusion of atmospheric oxygen into the yolk matrix, thereby markedly accelerating the autoxidation of unsaturated fatty acids [10]. An additional factor driving degradation is the natural microflora colonising the outer shell surface, predominantly represented by bacteria of the genera Pseudomonas and Micrococcus, as well as filamentous fungi [10]. During egg storage, these microorganisms are capable of synthesising and secreting extracellular lipolytic and oxidative enzymes which, upon penetrating through the shell pores into the yolk, catalyse triglyceride hydrolysis and secondary lipid oxidation [11,12]. Consequently, contemporary food technology is seeking innovative, non-contact methods to extend egg shelf life that effectively mitigate microbial contamination of the shell without adversely affecting the nutritional quality of the internal components. One of the most promising technologies in this regard is the application of gaseous ozone. As a potent oxidant with a redox potential of 2.07 V, ozone exhibits excellent biocidal properties against a broad spectrum of pathogens and spoilage microflora [13]. In the context of the proposed study, molecular stability refers to the preservation of the native chemical structure of lipid molecules and the prevention of their oxidative degradation during storage. The yolk matrix is a complex emulsion of low- and high-density lipoproteins (LDLs and HDLs), whose endogenous redox balance is naturally maintained by antioxidants such as α-tocopherol and carotenoids. However, there is a well-founded concern that contact with ozone may disrupt this balance. Ozone can react with unsaturated fatty acids via the Criegee mechanism, where the electrophilic addition of ozone to a carbon–carbon double bond forms a primary ozonide. This rapidly decomposes into reactive carbonyls and carbonyl oxides, driving further lipid peroxidation [14]. Additionally, reactive oxygen species (ROS) generated during this process can induce the oxidation of cholesterol, potentially yielding health-hazardous cholesterol oxidation products (COPs) such as 7-ketocholesterol and 7α/β-hydroxycholesterol [15,16].

While previous studies have extensively evaluated single, acute ozone exposures primarily for rapid shell decontamination, research thoroughly analysing the effect of long-term, cyclic treatment of intact eggs on internal lipid stability remains scarce. To address this knowledge gap, a study was undertaken to evaluate the impact of regular, cyclic ozonation of table eggs. The specific treatment regimen (5 ppm for 10 min, once weekly) was selected based on our preliminary trials, which demonstrated that this parameter combination provides sufficient antimicrobial efficacy on the shell surface without causing macroscopic damage to the cuticle layer. The primary aim was to assess the effect of this cyclic treatment on the fatty acid profile (FAME) and yolk cholesterol content during 5 weeks of storage, which serve as robust, fundamental indicators of lipid fraction preservation. The study also verified the research hypothesis, assuming that controlled ozonation at an effective dose—rather than accelerating oxidation—may paradoxically stabilize the yolk lipid fraction by preserving the internal physicochemical barriers of the egg and eliminating the enzymatic activity of the shell microflora.

## 2. Results and Discussion

### 2.1. Fatty Acid Profile of Fresh Egg Yolk and Its Changes During Storage (SFA, MUFA, and PUFA)

**Figure 1:**
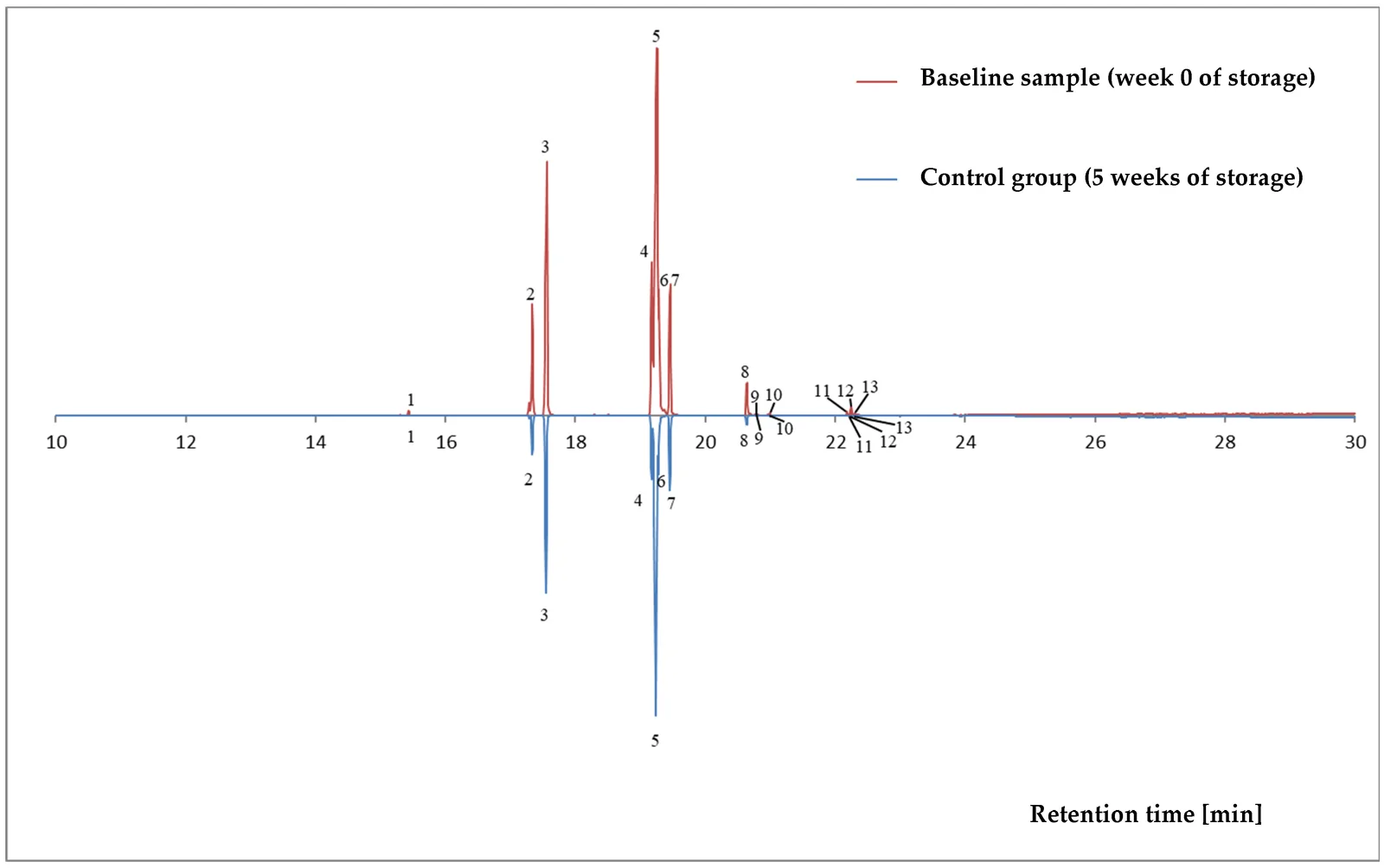
Comparison of the unstored sample (week 0) and the unozonated sample stored for 5 weeks. NOTE: Peak identification: 1: myristic acid; 2: palmitoleic acid; 3: palmitic acid; 4: linoleic acid; 5: oleic acid n-9; 6: oleic acid n-7; 7: stearic acid; 8: eicosa-5,8,11,14-tetraenoic acid; 9: eicosa-8,11,14,17-tetraenoic acid; 10: eicos-11-enoic acid; 11: eicosa-5,8,11,14,17-pentaenoic acid; 12: docosa-4,7,10,13,16,19-hexaenoic acid; 13: docosa-7,10,13,16,19-pentaenoic acid.

Chromatographic analysis of fresh egg yolk (week 0) revealed the presence of thirteen major fatty acids (Table 1, Figure 1). The dominant fraction comprised monounsaturated fatty acids (MUFAs), among which oleic acid n-9 (C_18:1, n-9_) exhibited the highest percentage share (47.57%), while oleic acid n-7 (C_18:1, n-7_) and palmitoleic acid (C_16:1, n-9_) accounted for 5.82% and 4.91%, respectively. The second most abundant fraction was saturated fatty acids (SFAs), predominantly represented by palmitic acid (C_16:0_; 21.92%) and stearic acid (C_18:0_; 8.74%). The proportion of myristic acid (C_14:0_) was negligible at 0.19%. Among polyunsaturated fatty acids (PUFAs), the highest contents were observed for linoleic acid (C_18:2_; 8.88%) and arachidonic acid (C_20:4, n-6_; 2.01%). Other long-chain fatty acids, including eicosapentaenoic acid (EPA, C_20:5, n-3_; 0.12%) and docosahexaenoic acid (DHA, C_22:6, n-3_; 0.41%), were present in smaller quantities (Table 1). This baseline fatty acid composition is largely representative of hen egg yolk, as confirmed by numerous studies on the lipid profile of eggs obtained from laying hens fed standard commercial diets [17,18].

In the control group (unozonated eggs), storage time exerted a highly statistically significant effect on the relative yolk fatty acid profile. Two-way analysis of variance (ANOVA) demonstrated significant changes in the relative percentage share for the majority of the analysed fatty acids, including saturated acids (myristic and stearic), monounsaturated oleic acid, and key polyunsaturated acids (linoleic, arachidonic, and DHA). Pearson’s simple correlation analysis confirmed that over time, a successive, linear decrease in the relative proportion of the most important unsaturated fatty acids occurred in the control group. This trend was most pronounced in the cases of myristic acid, eicos-11-enoic acid, and docosahexaenoic acid (DHA). A particularly marked relative decrease was observed for linoleic acid, the proportion of which dropped from an initial 8.88% to 5.68% at week 5 of storage, as well as for DHA, which underwent an almost complete reduction in its relative share towards the end of the study period (weeks 4–5), reaching trace levels (Table 1, Figure 1).

Conversely, an opposite trend was observed for saturated palmitic acid; its percentage share exhibited a positive correlation with storage time. It is crucial to emphasise that this trend does not indicate an increase in the absolute concentration of palmitic acid in the yolk [19]. Since the FAME results are expressed as the relative percentage of the total peak area, this phenomenon reflects a relative mathematical shift. As the highly oxidation-prone unsaturated fatty acids (which possess reactive double bonds) are postulated to undergo autoxidative processes, their relative peak areas decrease. Consequently, this proportional reduction mathematically increases the relative percentage share of the more stable, oxidation-resistant saturated fatty acid fraction. Although hen egg yolk is rich in native antioxidants (such as α-tocopherol, lutein, and zeaxanthin), we hypothesise that these are gradually depleted over time, which could contribute to the observed shifts in the relative PUFA proportions [20,21,22,23].

It is hypothesised that the diffusion of atmospheric oxygen penetrating through the micropores of the mineral shell, alongside oxygen dissolved in the albumen, may initiate the formation of alkyl and peroxyl radicals that attack the vulnerable double bonds in PUFAs (primarily in linoleic acid and DHA, which possesses as many as six double bonds). According to the literature, this mechanism leads to the formation of lipid hydroperoxides and their subsequent breakdown into short-chain aldehydes and ketones. While the observed decrease in the relative percentage share of the PUFA fraction in the control group is highly compatible with these oxidative processes, it must be explicitly stated that FAME analysis alone does not allow for direct confirmation of the occurrence or extent of lipid oxidation. The exact verification of this degradative pathway would require the direct determination of primary or secondary lipid oxidation products. However, since oxidation products were not directly quantified, this oxidative pathway remains a plausible mechanistic explanation rather than a confirmed chemical process in this study.

### 2.2. Effect of Regular Ozonation on the Stabilization of the Yolk Lipid Profile

Cyclic exposure of eggs to ozone (5 ppm for 10 min) was associated with the preservation of the relative proportion of yolk fatty acids. The two-way ANOVA revealed a highly significant time × ozonation interaction for the major polyunsaturated fatty acids (PUFAs), confirming that the evolution of the FAME profile fundamentally differed between the two treatments. Specifically, while the control group showed dynamic changes over time, a complete absence of statistically significant differences in the relative percentage share of the analysed fatty acids was demonstrated in the ozonated group across the entire 5-week experimental period. Furthermore, Pearson correlation analysis indicated that no statistically significant linear association was detected between storage time and the relative proportions of fatty acids in the ozonated group, which supports the observation of a preserved relative FAME profile without implying absolute stability. However, it is critical to emphasize that the fatty acid data reflect relative percentages of total FAME peak areas rather than absolute quantitative concentrations. While stable PUFA proportions indicate the absence of preferential degradation among specific fatty acid classes, relative chromatographic profiling alone cannot directly demonstrate or rule out oxidative degradation.

**Figure 2:**
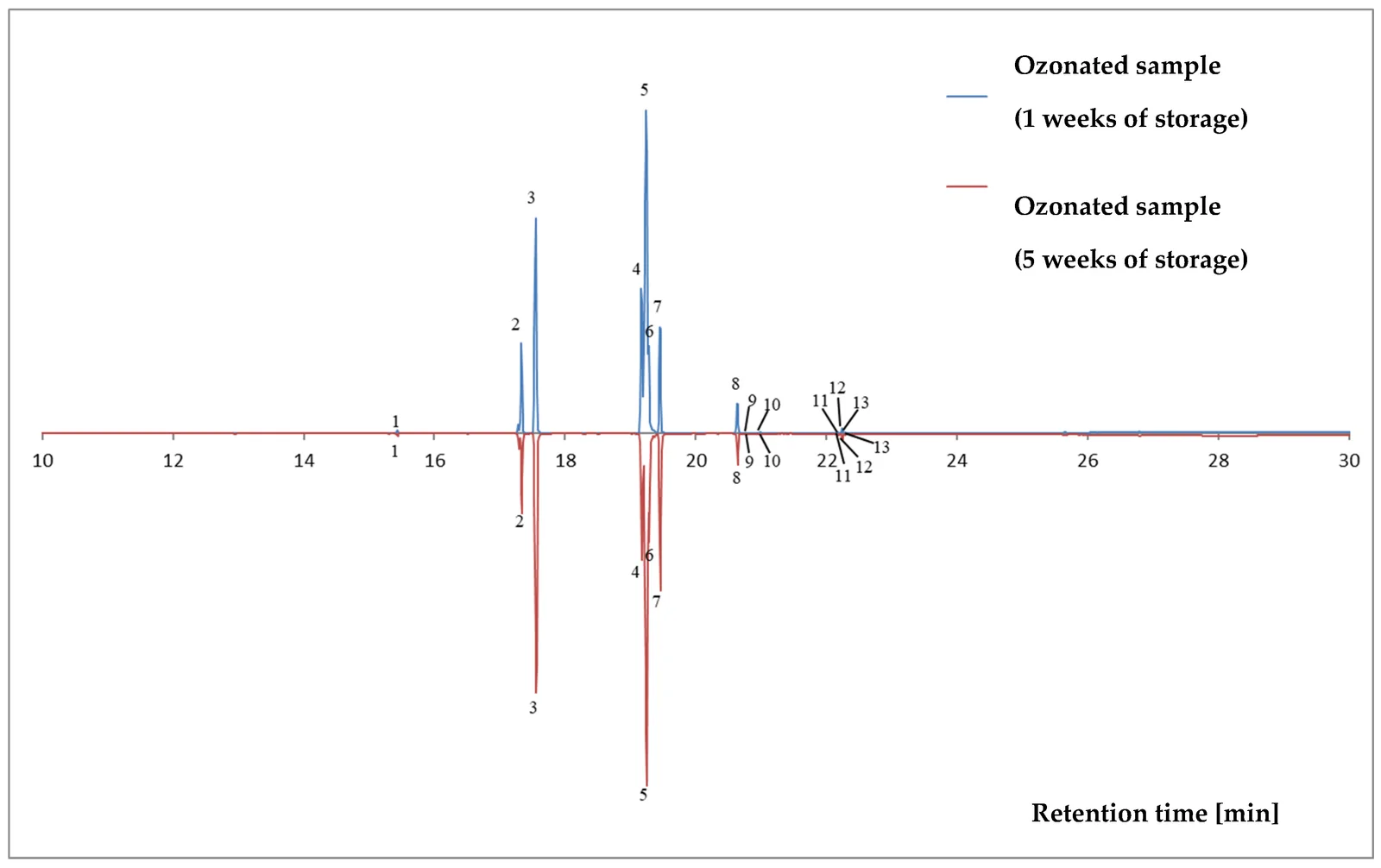
Comparison of ozonated samples stored for 1 and 5 weeks. NOTE: Peak identification: 1: myristic acid; 2: palmitoleic acid; 3: palmitic acid; 4: linoleic acid; 5: oleic acid n-9; 6: oleic acid n-7; 7: stearic acid; 8: eicosa-5,8,11,14-tetraenoic acid; 9: eicosa-8,11,14,17-tetraenoic acid; 10: eicos-11-enoic acid; 11: eicosa-5,8,11,14,17-pentaenoic acid; 12: docosa-4,7,10,13,16,19-hexaenoic acid; 13: docosa-7,10,13,16,19-pentaenoic acid.

**Figure 3:**
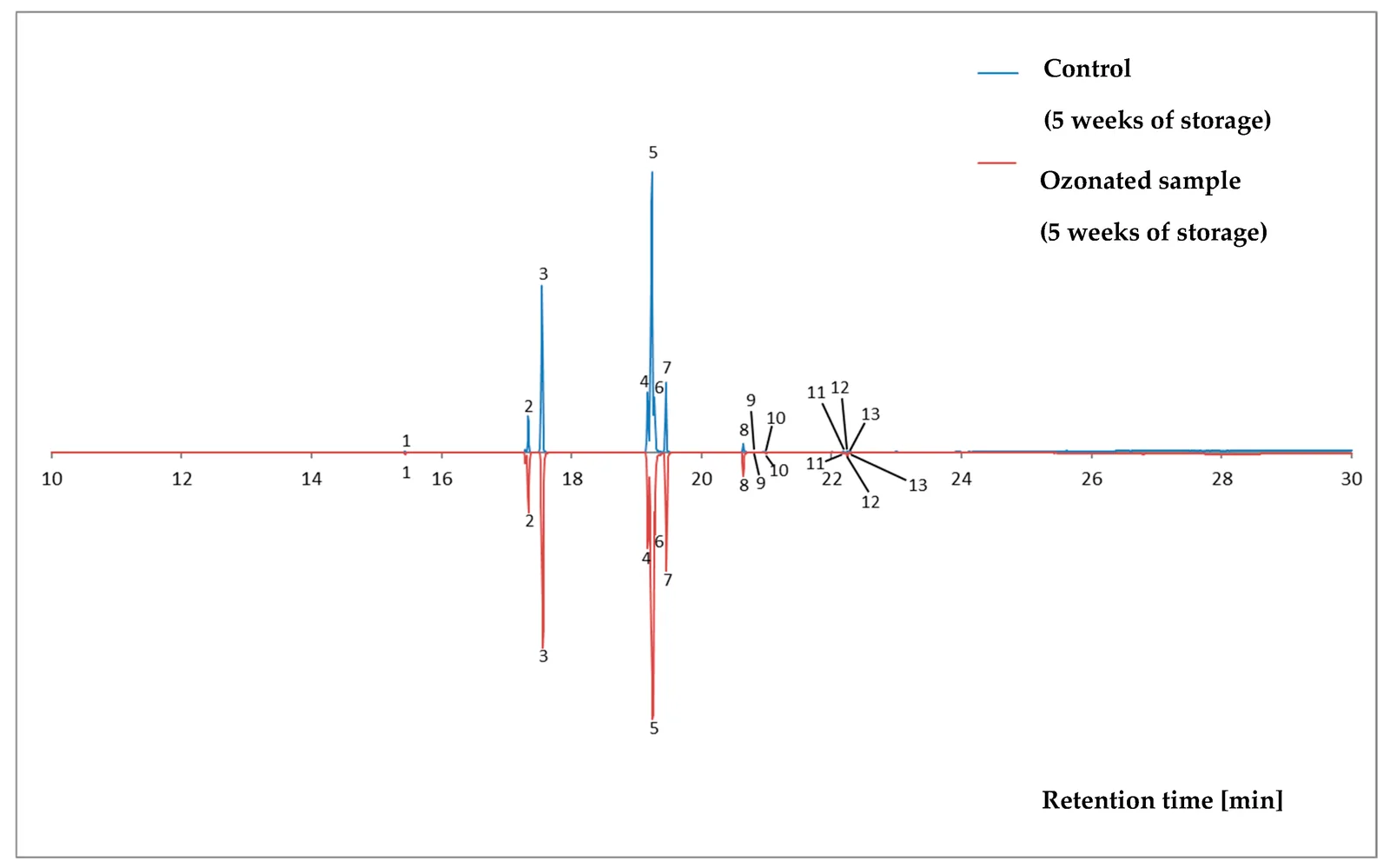
Comparison of control and ozonated samples stored for 5 weeks. NOTE: Peak identification: 1: myristic acid; 2: palmitoleic acid; 3: palmitic acid; 4: linoleic acid; 5: oleic acid n-9; 6: oleic acid n-7; 7: stearic acid; 8: eicosa-5,8,11,14-tetraenoic acid; 9: eicosa-8,11,14,17-tetraenoic acid; 10: eicos-11-enoic acid; 11: eicosa-5,8,11,14,17-pentaenoic acid; 12: docosa-4,7,10,13,16,19-hexaenoic acid; 13: docosa-7,10,13,16,19-pentaenoic acid.

A comparison of the chromatograms clearly illustrates this relationship: while in the control group the peaks representing PUFAs underwent a drastic reduction (Figure 1), the profile of the ozonated sample after 5 weeks of storage (Figure 2 and Figure 3) remained virtually identical to that of the fresh sample. Given the high redox potential of ozone (2.07 V) [24] and its capacity for direct Criegee ozonolysis reactions with double bonds in unsaturated lipids [25], its apparent stabilizing influence on the relative FAME profile appears paradoxical. Theoretically, this gas should rapidly initiate pro-oxidative processes and autoxidation. However, in the absence of direct primary oxidation markers such as peroxide value (PV) or conjugated dienes, this stability should not be interpreted as definitive proof of an active protective antioxidant mechanism. Instead, the explanation for this phenomenon lies primarily in the barrier properties of the physical structures of the hen egg and the specific mechanism of action of low, effective doses of gaseous ozone.

A vital role is played by the barrier effect of the egg structures, which consists of the physical isolation of the yolk from the external environment by the mineral shell, two shell membranes, the albumen (egg white) layer, and the vitelline membrane. Literature reports unequivocally indicate that ozone applied in the gaseous phase at low concentrations (up to 5–6 ppm) reacts almost exclusively at the shell surface and does not penetrate into the yolk interior [26]. This prevents direct contact between O_3_ molecules and yolk lipids, thereby avoiding ozone-induced oxidative degradation of double bonds directly within the yolk.

In addition to this direct physical barrier, literature suggests that a potential element of the protective mechanism may be linked to the preservation of the structural integrity of the albumen and the vitelline membrane. Under natural conditions, egg ageing is associated with the loss of carbon dioxide (CO_2_) through the shell pores, resulting in an increase in albumen pH [27]. Such an increase in environmental alkalinity is known to lead to the depolymerisation of the ovomucin–lysozyme complex, thinning of the thick albumen gel, and weakening of the vitelline membrane structure, which facilitates oxygen diffusion [28]. While our study did not directly determine shell permeability, CO_2_ loss, albumen pH, albumen viscosity, vitelline membrane integrity, or oxygen availability, it is hypothesised based on previous reports that ozonation might limit water evaporation and CO_2_ loss by modifying the cuticle. These proposed physical and barrier-related mechanisms were not directly evaluated and must be clearly distinguished from the experimentally demonstrated findings (such as the FAME profile stability). Therefore, these interpretations should be viewed strictly as speculative explanations requiring future direct verification. If these natural physical barriers are indeed preserved by the treatment, it could theoretically restrict oxygen availability to the yolk lipids, providing a plausible explanation for the observed stability of the relative PUFA profile [29].

Furthermore, the stabilisation of the relative FAME profile could hypothetically be linked to the superficial antimicrobial action of ozone. The shell surface of fresh eggs is naturally colonised by microbiota that can secrete extracellular lipolytic and oxidative enzymes during storage [30]. While microbial load and enzymatic activity were not directly evaluated in the present study, ozone is widely recognised for its efficacy in reducing shell microbiota [29]. Because microbiological parameters were beyond the scope of our direct measurements, the potential involvement of microbial enzymes in yolk lipid degradation remains entirely hypothetical. It is biologically plausible that this superficial antimicrobial action might limit the potential penetration of microbial enzymes into the egg interior, thereby serving as an additional, purely theoretical contribution to the preservation of yolk lipids during storage.

### 2.3. Cholesterol Content and Its Stability During Storage and Ozonation

The baseline cholesterol concentration in fresh yolk was 16.89 mg g^−1^ (Table 2). Over the 5-week storage period, no statistically significant differences in yolk cholesterol content were observed in either the control or the ozonated group compared to the initial value (*p* > 0.05 for all comparisons, Table 2).

The slight numerical increase in average cholesterol concentration values noted at weeks 4 and 5 of storage (up to 20.35 mg g^−1^ for the control group and 18.48 mg g^−1^ for the ozonated group) was not statistically significant. It is hypothesised that this phenomenon may result from the natural loss of water from the yolk (migration into the albumen driven by osmotic pressure and evaporation through the shell pores), which could lead to an apparent, physical concentration of the yolk dry matter components [29]. However, because moisture content, yolk mass, and overall egg weight loss were not directly determined in the present study, this explanation remains a biologically plausible hypothesis rather than a demonstrated effect. Furthermore, Pearson correlation analysis indicated that no statistically significant linear association was detected between storage duration and total cholesterol content in either analysed group (Table 3).

Cholesterol is an organic sterol susceptible to oxidation, particularly under the influence of thermal factors, UV radiation, or potent pro-oxidants, leading to the formation of health-hazardous cholesterol oxidation products (COPs) [21]. The fact that cyclic ozonation did not induce a statistically significant decline in total cholesterol content over the 5-week period indicates that the treatment did not significantly affect this primary parameter. However, it must be explicitly stated that the determination of total cholesterol alone does not allow for the assessment of specific COP formation. Therefore, while the absence of quantitative changes in total cholesterol is a relevant finding regarding the macroscopic stability of the yolk matrix, it does not completely exclude the potential occurrence of cholesterol oxidation. Consequently, further specialized chromatographic studies directly determining COP concentrations are required to fully confirm the chemical safety of this technology.

## 3. Materials and Methods

### 3.1. Biological Material and Experimental Design

The research material consisted of 300 table eggs obtained from Isa Brown laying hens kept in a cage housing system, fed a standard commercial layer diet. On the first day of the experiment, all eggs were randomly divided into two equal treatment groups: a control group (*n* = 150) and an ozonated group (*n* = 150). The eggs were collected on the day of laying and classified as size M with an average weight of 55 g. Prior to the experiment, strict inclusion criteria were applied: all eggs underwent visual inspection and candling to exclude specimens with micro-cracks, visible surface contamination, or cuticle damage that could unseal the shell barriers and alter ozone penetration. During the 5-week experimental period, the eggs were stored pointed end down under controlled conditions at a temperature of 20 °C, relative humidity of 50%, with standard natural ventilation and in the absence of light. As justified by preliminary antimicrobial screening, the experimental group was regularly exposed once a week to ozone at a concentration of 5 ppm for 10 min. The ozonation process was conducted under constant, controlled ambient temperature and relative humidity using a Korona L ozone generator with an output of 10 g h^−1^ (Koronaozon, Piotrków Trybunalski, Poland). The gas concentration was continuously monitored using a UV-106M Medium Range Ozone Analyzer (Ozone Solutions, Hull, IA, USA). The remaining eggs were not exposed to the gas and served as the control group.

Samples were collected at weekly intervals, defining six time points: fresh eggs (week 0) and eggs stored for 1, 2, 3, 4, and 5 weeks, respectively. At each time point, selected eggs were broken and separated into albumen and yolk fractions. Individual components were weighed on a RADWAG WBT 200 analytical balance (Radwag, Radom, Poland) and pooled into homogeneous samples (each pooled sample consisted of three yolks originating from the same treatment group). Albumen and yolk were pooled separately to avoid cross-contamination. The prepared yolk and albumen masses were transferred to cryogenic tubes and frozen in a low-temperature laboratory freezer (Sanyo Electric Co., Ltd., Osaka, Japan) at −67 °C until further analysis.

### 3.2. Sample Preparation for Fatty Acid Profile Analysis

The yolk was separated from the albumen using a separator. The intact yolk was gently rolled on sterile filter paper to remove residual albumen. Subsequently, the vitelline membrane was punctured with a sterile scalpel, and the internal yolk contents were collected into sterile containers. To prepare representative laboratory samples, three individual yolks were pooled and homogenized together to form a single composite sample. Homogenization was performed using a vortex mixer at 2000 rpm (Vortex-Genie 2, Scientific Industries, OHAUS Corporation, Parsippany, NJ, USA) for 2 min. Consequently, 4 independent composite samples (*n* = 4) were effectively obtained for each treatment at each time point, and this composite sample was considered the independent experimental unit for all subsequent analyses. The homogenised composite samples were immediately stored at −67 °C. For the isolation and derivatisation of fatty acids, 0.25 g of homogenised yolk from the laboratory samples was weighed into disposable 15 mL polypropylene Falcon conical centrifuge tubes. A 12.5 mL volume of a chloroform/methanol mixture (Chempur, Piekary Śląskie, Poland) in a 2:1 ratio (*v*/*v*) was added to the sample and vigorously mixed using a vortex mixer to achieve complete extraction homogeneity. Next, 2.5 mL of a 0.58% aqueous solution of sodium chloride (Chempur, Piekary Śląskie, Poland) was added. Phase separation was accelerated by centrifugation in a Sigma 3-16PK universal laboratory centrifuge (Sigma Laborzentrifugen GmbH, Osterode am Harz, Germany) at 4500 rpm (approx. 2400× *g*) for 3.5 min. Aliquots of 2 mL were collected from the lower organic phase into glass tubes and evaporated to dryness under a gentle stream of nitrogen at 65 °C using a VLM nitrogen evaporator (VLM GmbH, Bielefeld, Germany).

The dry residue was subjected to saponification by adding 1.5 mL of a 0.5 M solution of sodium hydroxide (Honeywell, Charlotte, NC, USA) in methanol and heating at 80 °C for 20 min in a MyBath 4L water bath (Model B2000-4-E; Benchmark Scientific, Sayreville, NJ, USA). After cooling to room temperature, transesterification was carried out by adding 1.5 mL of boron trifluoride (Sigma-Aldrich, St. Louis, MO, USA) in methanol solution (concentration 49.0–53.0%), followed by reheating in the water bath at 80 °C for 10 min. The resulting fatty acid methyl esters (FAMEs) were extracted by adding 1 mL of n-hexane (95% purity; Sigma-Aldrich, USA) and vigorously shaking. To improve phase separation, the samples were chilled in a chest freezer (Whirlpool Corp., Benton Harbor, MI, USA) at −21 °C for 20 min and subsequently centrifuged (4500 rpm, 3.5 min). The upper hexane layer containing FAMEs was collected and transferred to glass chromatographic vials. Identification and calibration were based on comparison with the analytical standard of fatty acid methyl esters, PUFA No. 3 from menhaden oil (Supelco, Bellefonte, PA, USA; Cat. No. 47085-U).

### 3.3. Sample Preparation for Cholesterol Content Analysis

Representative and laboratory samples were prepared according to the procedure described in Section 3.1. Aliquots of homogenised yolk weighing 0.125 g were transferred into polypropylene centrifuge tubes. Saponification was initiated by adding 2 mL of 96% ethanol (analytical grade; Honeywell) and 0.25 mL of a 60% aqueous solution of potassium hydroxide (Chempur, Piekary Śląskie, Poland). The mixture was vortexed and heated in a water bath at 70 °C for 1 h. Following thermal treatment, the samples were vortexed again and cooled to room temperature. To extract the unsaponifiable fraction, 2 mL of a 1% NaCl solution (Chempur, Piekary Śląskie, Poland) and 4 mL of n-hexane (Chempur, Piekary Śląskie, Poland) were added to the samples, followed by intensive mechanical shaking for 10 min. The upper organic layer was collected. The remaining aqueous phase was re-extracted with another 4 mL portion of n-hexane under identical conditions (10 min of shaking). Both hexane fractions were combined and evaporated to dryness under a nitrogen stream using a VLM evaporator. The dry residue was quantitatively redissolved in 2 mL of *n*-hexane and transferred to chromatographic vials. Quantitative calibration was performed using a pure cholesterol standard (Sigma Grade, purity 99%; Sigma-Aldrich).

### 3.4. Gas Chromatography-Mass Spectrometry (GC-MS) Analysis

Qualitative analysis of the FAME profile and quantitative determination of cholesterol content were performed using a gas chromatography system interfaced with mass spectrometry (GC-MS), consisting of a Varian 450-GC gas chromatograph coupled to a Varian 240-MS mass spectrometer (Varian Inc., Walnut Creek, CA, USA).

#### 3.4.1. Determination of Fatty Acid Profile (FAME)

Chromatographic separation of FAME was achieved on a VF-5ms capillary column (30 m × 0.25 mm ID, 0.25 µm film thickness). To ensure high reproducibility, the following chromatographic conditions were strictly maintained: Helium was used as the carrier gas at a constant flow rate of 1.0 mL min^−1^. The PTV 1079 injector was maintained at 200 °C and operated in splitless mode, with an injection volume of 1 µL. The column oven temperature program started at 50 °C, increased to 300 °C at a rate of 10 °C min^−1^, and was then held isothermally at 300 °C for 20 min. Mass spectra were recorded in electron ionisation (EI) mode at an ionising energy of 70 eV in full-scan mode covering the mass acquisition range of *m*/*z* 50–500.

Regarding the quantification criteria, FAME identification was primarily carried out by comparing retention times with the commercial standard reference mixture (PUFA No. 3 from menhaden oil), which explicitly contained all 13 of the identified fatty acids. Additionally, the mass spectra of the detected compounds were cross-verified against the NIST mass spectral library to definitively confirm their structural identity. Peak integration was performed using the Workstation Toolbar software package. The results were not expressed as absolute concentrations but rather as the relative percentage share (%) of individual fatty acids, calculated from the total FAME peak area.

#### 3.4.2. Determination of Cholesterol Content

Cholesterol analysis was performed using the same VF-5ms capillary column and under identical mass spectrometer operating conditions (EI, 70 eV, *m*/*z* range 50–500). Chromatographic separation was carried out under the following oven temperature conditions: an initial isothermal temperature of 265 °C (held for 20 min), followed by a ramp to 300 °C at a rate of 10 °C min^−1^ and a final hold at this temperature for 5 min. The injector temperature was set to 280 °C, and the injection volume was 1 µL in splitless mode. A calibration curve was constructed based on the analysis of cholesterol standard solutions in n-hexane at concentrations of 0.75, 1.0, 1.5, and 2.0 mg mL^−1^ (a range tailored to the physiological cholesterol content in egg yolk). Quantitative analysis was performed using the external standard method. All instrumental measurements for each composite sample were performed in triplicate. The method demonstrated satisfactory linearity within the tested range, with a coefficient of determination *R*^2^ = 0.998. The limit of detection (LOD) and limit of quantification (LOQ) were determined to be 0.1 mg g^−1^ and 0.3 mg g^−1^, respectively. The analytical method precision, expressed as relative standard deviation (RSD), was 3.2%, and the mean recovery was 95.5%.

### 3.5. Statistical Analysis

The obtained data were subjected to statistical analysis using STATISTICA software (version 12; StatSoft Inc., Tulsa, OK, USA). The composite yolk sample was strictly defined as the experimental unit for all statistical tests (*n* = 4 per treatment group at each time point). Prior to analysis, the normality of data distribution was verified using the Shapiro–Wilk test, while the homogeneity of variances was evaluated using Levene’s test. To examine the effects of storage duration (Factor I) and the ozonation process (Factor II), as well as the interaction between these factors (storage time–ozonation), a two-way analysis of variance (two-way ANOVA) was applied. The significance of differences between mean values in individual groups and time points was verified using Tukey’s post hoc test (HSD). All statistical analyses were performed at a significance level of *p* < 0.05. Results are presented as mean values and standard deviations (mean ± *SD*).

## 4. Conclusions

This study demonstrated that over five weeks of storage, the control group of table eggs exhibited significant changes in the relative profile of several fatty acids, notably including a relative decrease in the proportion of polyunsaturated fatty acids (PUFAs) such as docosahexaenoic acid (DHA). Conversely, the group subjected to cyclic gaseous ozonation (5 ppm for 10 min) showed an absence of statistically significant differences in the relative FAME profile throughout the entire storage period. Furthermore, the total cholesterol content in the yolk was not significantly affected by either prolonged storage or the ozonation process.

However, because fatty acids were evaluated solely as relative area percentages of FAME, these findings cannot directly confirm absolute oxidative stability or the complete absence of lipid degradation. While these findings indicate that cyclic ozonation is associated with a preserved relative FAME profile and stable total cholesterol levels under the evaluated storage conditions, any proposed protective mode of action remains a speculative working hypothesis. Tracking the precise physicochemical pathways and assessing primary (e.g., peroxide value, conjugated dienes) and secondary oxidation markers, as well as cholesterol oxidation products (COPs), represent essential requirements rather than merely valuable directions for dedicated future research. Consequently, further specialized studies are required to fully verify the chemical stability underlying mechanistic pathways and detailed oxidative status of the yolk components.

## Figures and Tables

**Table 1:** Determined profile of fatty acids in the egg yolk during the experiment (% content).

Storage Time	C_14_H_28_O_2_ (Myristic Acid)		C_16_H_32_O_2_ (Palmitic Acid)			C_16_H_30_O_2_ (Palmitoleic Acid n-9)		C_18_H_36_O_2_ (Stearic Acid)		C_18_H_34_O_2_ (Oleic Acid n-9)		C_18_H_34_O_2_ (Oleic Acid n-7)			C_18_H_32_O_2_ (Linoleic Acid)	
	Control (% of Total)	Ozonated (% of Total)	Control (% of Total)	Ozonated (% of Total)		Control (% of Total)	Ozonated (% of Total)	Control (% of Total)	Ozonated (% of Total)	Control (% of Total)	Ozonated (% of Total)	Control (% of Total)		Ozonated (% of Total)	Control (% of Total)	Ozonated (% of Total)
0 weeks	0.19 ^Aa^ ± 0.107		21.92 ^Aa^ ± 1.132			4.910 ^Aa^ ± 3.153		8.74 ^Aa^ ± 2.038		47.566 ^Aa^ ± 9.384		5.82 ^Aa^ ± 2.552			8.875 ^Aa^ ± 2.546	
1 week	0.13 ^ca^ ± 0	0.15 ^A^* ± 0.08	23.77 ^a^ ± 1.75	23.232 ^A^ ± 3.37		4.462 ^a^ ± 1.59	4.16 ^A^ ± 2.50	8.46 ^a^ ± 1.71	7.170 ^A^ ± 3.12	47.436 ^a^ ± 5.38	50.66 ^A^ ± 15.56	6.32 ^a^ ± 0.35		5.19 ^A^ ± 3.44	8.23 ^a^ ± 1.87	9.42 ^A^ ± 4.89
2 weeks	0.00 ^b^* ± 0	0.08 ^A^* ± 0.06	25.35 ^a^ ± 2.45	28.269 ^A^ ± 12.71		1.999 ^b^* ± 0.09	5.65 ^A^* ± 0.61	4.81 ^b^* ± 0.78	9.376 ^A^* ± 1.15	68.518 ^a^ ± 6.20	46.37 ^A^ ± 4.64	2.00 ^a^ ± 1.41		6.52 ^A^ ± 0.95	3.83 ^b^* ± 1.41	10.64 ^A^* ± 0.83
3 weeks	0.19 ^a^* ± 0.05	0.11 ^A^* ± 0.03	22.20 ^a^ ± 2.45	24.075 ^A^ ± 3.01		5.073 ^a^ ± 0.78	5.14 ^A^ ± 2.29	8.80 ^a^ ± 1.58	7.463 ^A^ ± 2.19	45.650 ^a^ ± 6.03	49.72 ^A^ ± 9.40	7.54 ^a^ ± 0.82		6.67 ^A^ ± 1.42	10.70 ^c^* ± 2.35	8.25 ^A^* ± 3.11
4 weeks	0.15 ^ab^ ± 0.01	0.08 ^A^* ± 0.09	21.51 ^a^ ± 0.70	26.656 ^A^ ± 6.44		5.995 ^a^ ± 0.61	6.11 ^A^ ± 0.39	8.89 ^a^ ± 0.28	9.827 ^A^ ± 0.67	42.183 ^a^ ± 0.81	54.49 ^A^ ± 15.32	6.28 ^a^ ± 3.69		3.84 ^A^ ± 4.48	12.44 ^d^ ± 0.12	11.30 ^A^ ± 0.02
5 weeks	0.00 ^b^* ± 0	0.15 ^A^* ± 0.10	26.12 ^a^ ± 5.40	22.737 ^A^ ± 1.83		2.990 ^c^* ± 1.61	4.11 ^A^* ± 3.51	6.49 ^ab^* ± 2.31	8.023 ^A^ ± 3.51	55.052 ^a^ ± 7.52	64.43 ^A^ ± 11.65	5.07 ^a^ ± 3.16		7.00 ^A^ ± 1.24	5.68 ^be^* ± 2.18	9.33 ^A^* ± 1.84
**Storage time**	**C_20_H_38_O_2_ (Eicos-11-enoic acid)**		**C_20_H_32_O_2_ (eicosa-8,11,14,17-tetraenoic acid)**				**C_20_H_32_O_2_ (eicosa-5,8,11,14-tetraenoic acid)**		**C_20_H_30_O_2_ (eicosa-5,8,11,14,17-pentaenoic acid)**		**C_22_H_34_O_2_ (docosa-7,10,13,16,19-pentaenoic acid)**				**C_22_H_32_O_2_ (docosa-4,7,10,13,16,19-hexaenoic acid)**	
	**Control (% of total)**	**Ozonated (% of total)**	**Control (% of total)**		**Ozonated (% of total)**		**Control (% of total)**	**Ozonated (% of total)**	**Control (% of total)**	**Ozonated (% of total)**	**Control (% of total)**		**Ozonated (% of total)**		**Control (% of total)**	**Ozonated (% of total)**
0 weeks	0.06 ^Aa^ ± 0.043		0.02 ^Aa^ ± 0.017				2.01 ^Aa^ ± 0.351		0.12 ^Aa^ ± 0.089		0.08 ^Aa^ ± 0.06				0.41 ^Aa^ ± 0.21	
1 week	0.03 ^A^ ± 0.04	0.03 ^a^ ± 0.03	Trace		0.98 ^b^* ± 0.86		2.01 ^A^* ± 0.27	0.14 ^a^* ± 0.17	0.03 ^A^ ± 0.04	0.03 ^a^ ± 0.031	Trace		0.98 ^b^* ± 0.86		2.01 ^A^* ± 0.27	0.14 ^a^* ± 0.17
2 weeks	0.05 ^A^ ± 0.07	0.00 ^a^ ± 0	Trace		Trace ^c^*		1.23 ^A^* ± 0.60	0.00 ^b^* ± 0	0.05 ^A^ ± 0.07	0.00 ^a^ ± 0	Trace		Trace ^c^*		1.23 ^A^* ± 0.60	0.00 ^b^* ± 0
3 weeks	0.04 ^A^ ± 0.04	Trace	Trace		1.43 ^d^* ± 0.75		1.94 ^A^* ± 0.21	0.24 ^c^* ± 0.16	0.04 ^A^ ± 0.04	Trace	Trace		1.43 ^d^* ± 0.75		1.94 ^A^* ± 0.21	0.24 ^c^* ± 0.15
4 weeks	0.05 ^A^ ± 0.03	0.01 ^a^ ± 0.004	0.02 ^A^ ± 0.12		1.93 ^a^* ± 0.12		1.00 ^A^* ± 0.03	0.15 ^a^* ± 0.03	0.05 ^A^ ± 0.03	0.01 ^a^ ± 0.01	0.02 ^A^ ± 0.12		1.93 ^a^* ± 0.12		1.00 ^A^* ± 0.03	0.15 ^a^* ± 0.03
5 weeks	trace	Trace	Trace		1.05 ^b^* ± 0		1.24 ^A^* ± 0.91	Trace ^b^*	trace	Trace	Trace		1.05 ^b^* ± 0		1.24 ^A^* ± 0.90	Trace ^b^*

NOTE: Values in the control columns followed by the same lowercase letters, and values in the ozonated columns followed by the same uppercase letters, are not statistically significant according to Tukey’s HSD test (α = 0.05). Values marked with an asterisk (*) within rows for a given week are significantly different between treatments according to Student’s *t*-test (α = 0.05). Trace—trace amounts.

**Table 2:** Cholesterol content depending on storage duration.

Storage Time	Sample Type	Mean Cholesterol Content in Yolk (mg g^−1^)	Standard Deviation	*p*
0 weeks	Baseline sample	16.89	1.6875	-
1 week	Control	18.67	3.5565	0.104
	Ozonated	16.73	1.5996	0.249
2 weeks	Control	16.81	1.9318	0.376
	Ozonated	17.71	1.2680	0.362
3 weeks	Control	16.46	1.5220	0.507
	Ozonated	16.74	2.0826	0.797
4 weeks	Control	19.80	6.6564	0.226
	Ozonated	24.19	11.9309	0.450
5 weeks	Control	20.35	7.2153	0.109
	Ozonated	18.48	3.3166	0.572

**Table 3:** Calculated correlation coefficients between cholesterol content and storage duration.

Sample Type	Linear Correlation Coefficient *r* (X,Y)	*p*
Control	0.23	0.165
Ozonated	0.24	0.202

## Data Availability

All data are included in the article.
